# A fifteen-year retrospective analysis of varicocele embolization: evaluating success, recurrence rates and embolic agents

**DOI:** 10.1186/s42155-025-00575-6

**Published:** 2025-07-23

**Authors:** Meadhbh Ni Mhiochain de Grae, Maha Al-khattab, Amor Alkadhimi, Maia Springael, Gerry O’Sullivan

**Affiliations:** 1 Department of Interventional Radiology, Galway University Hospital, Galway, Ireland; 2 Tallaght University Hospital, Dublin, Ireland

**Keywords:** Varicocele, Embolization, Infertility, Male, Vascular surgical procedures, Percutaneous procedures, Radiology, Interventional, Treatment outcome, Recurrence, Fertility, Sclerosing solutions

## Abstract

**Introduction:**

A varicocele is a venous dilatation due to valvular incompetence within the pampiniform plexus, affecting 10–20% of the population and found in 40% of men with primary infertility (Hum Reprod Update 7(1):59-64, 2001, Cochrane Database System Rev (3), 2004, Curr Urol 6(1):33-6, 2012, World J Men's Health 37(1):4, 2019). Varicocele associated pain occurs in 2–10% of cases (Hum Reprod Update 7(1):59-64, 2001, SpringerPlus 4:1-5, 2015). Treatment options include conservative management, percutaneous embolization, or surgery (Urology 72(1):77-80, 2008). In the literature, percutaneous embolization has a technical failure rate ranging from 0 to 13.9% and recurrence rates of around 13% (Cochrane Database System Rev 4(4):CD000479, 2021).

This study evaluates the success and recurrence of percutaneous varicocele embolizations over fifteen years and compares the embolic materials used.

**Methods:**

This was a retrospective study of all adult patients who underwent varicocele embolization performed from April 2008 to February 2023 in two tertiary centres. Data collected included patient age, procedure date, access site, side of occurrence, previous interventions, treatment method, need for re-intervention, and recurrence rates. We defined technical success as successful access to the gonadal vein and embolization of same with coil/sclerosant. We assessed clinical success through follow-up telephone consultations and ultrasound.

**Results:**

The technical and clinical success rate was 96% and 93.75%, respectively. Of 225 patients, 3.12% had prior failed surgeries, all were treated successfully with IR, and only 0.89% required further surgical intervention. Patients reported recurrence rate of 25% of cases during telephone follow-up. However, the confirmed actual recurrence rate based on ultrasound was only 6.25%. The complication rate was low (1.78%), with no major events. Among patients treated for subfertility, 51.35% achieved successful conception following percutaneous embolization. Outcomes did not significantly differ based on the type of embolic material used.

**Conclusion:**

Percutaneous embolization is a safe, effective, and durable treatment for varicocele, demonstrating high technical and clinical success regardless of embolic material used with a low recurrence rate over long-term follow-up. It remains effective even in cases of prior failed surgical repair and is associated with promising fertility outcomes. These findings support embolization as a first-line treatment in varicocele management.

## Introduction

A varicocele is a venous dilatation due to valvular incompetence within the pampiniform plexus [1]. Varicoceles are seen in 10–20% of the population and are usually left-sided [2, 3]. Varicoceles are the most commonly identifiable abnormality found in men evaluated for infertility [1]. However, there are a myriad of other causes. Testicular varicoceles are identified in 40% of men with primary infertility and 80% of men with secondary infertility [4]. Two to ten per cent of men with varicoceles complain of scrotal pain or discomfort [1, 5].

Although incompletely understood, the pathophysiology of varicoceles is likely to involve incompetent valves in the spermatic veins, leading to the reflux of warm abdominal blood into the scrotum and a subsequent rise in scrotal temperature [1]. Evidence suggests that varicoceles have a progressive, deleterious effect on testicular size, function and fertility over time [6].

Varicocele diagnosis is clinical. Based on clinical findings, there are three arbitrary grades of varicoceles: large varicoceles are visible, medium varicoceles are palpable and small varicoceles are only palpable during a Valsalva manoeuvre [1]. Subclinical varicoceles can be visualized on ultrasound. However, there is no consensus on the optimal criteria for ultrasound diagnosis. Various classifications are available; thus, preference depends on the individual clinician [8].

Treatment options for testicular varicoceles include conservative management, percutaneous embolization or surgical varicolectomy. Percutaneous embolization as a varicocele treatment has been used since the late 1970 s with excellent outcomes [9]. Technical failure is thought to occur in up to 13.9% of cases. [10] Percutaneous treatments have lower complication rates than surgical alternatives (RR 0.63) [11]. Specifically, testicular loss from arterial injury occurs in 1% of surgical cases, whereas no instances of this complication have been reported with percutaneous interventions [12]. Patients are usually discharged on the same day following percutaneous intervention [13].

Many different embolic agents are available for percutaneous embolization of varicoceles. These include liquid embolics, embolization coils and occlusion devices [14]. However, due to significant heterogeneity studies and small sample sizes, no consensus has been reached on the optimum embolic materials for varicocele embolization [14]. Currently, the choice of embolic agent depends mainly on operator preference.

Our study aimed to assess the efficacy and recurrence rates of all percutaneous varicocele embolizations performed at two centres over fifteen years, with a minimum follow-up duration of 18 months. The outcomes associated with the various embolic agents used were also compared.

## Methods

This retrospective cohort study was conducted at two tertiary referral interventional radiology centres. This manuscript adheres to the STROBE guidelines for cohort studies. Consecutive adult patients who underwent varicocele embolization performed from April 2008 to February 2023 were included in this retrospective cohort study. Eligible patients were identified via a retrospective review of procedural records and radiology reports. All consecutive adult patients undergoing varicocele embolization during the study period were included. No formal sample size calculation was performed due to the study's retrospective nature. Bilateral procedures were treated as a single entry. A single operator performed all procedures with over 20 years of experience in varicocele embolizations.

Descriptive statistics were used to summarize patient demographics and procedural characteristics. Categorical data were presented as frequencies and percentages. Data analysis was performed using Microsoft Excel 2021 (version 16) and IBM SPSS Statistics (version 30).

Data points collected included age at the time of the procedure, date of procedure, access site used (right internal jugular veins vs. right common femoral vein), site of occurrence, previous failed interventions, type of embolic agent used, need for re-intervention and recurrence rate.

Technical success was measured as successful access to the gonadal vein and the successful insertion of coil/sclerosant with abolition of flow in that gonadal vein. This definition was applied uniformly to both right- and left-sided varicoceles.

Subjective clinical success was assessed through telephone consultations, which were also used to determine rates of successful fertility and subjective recurrence. Those who self-reported varicocele recurrence were offered a repeat ultrasound to assess for true recurrence. A summary of the items included in the telephone consultation questionnaire is detailed in Table 1.

**Table 1:**
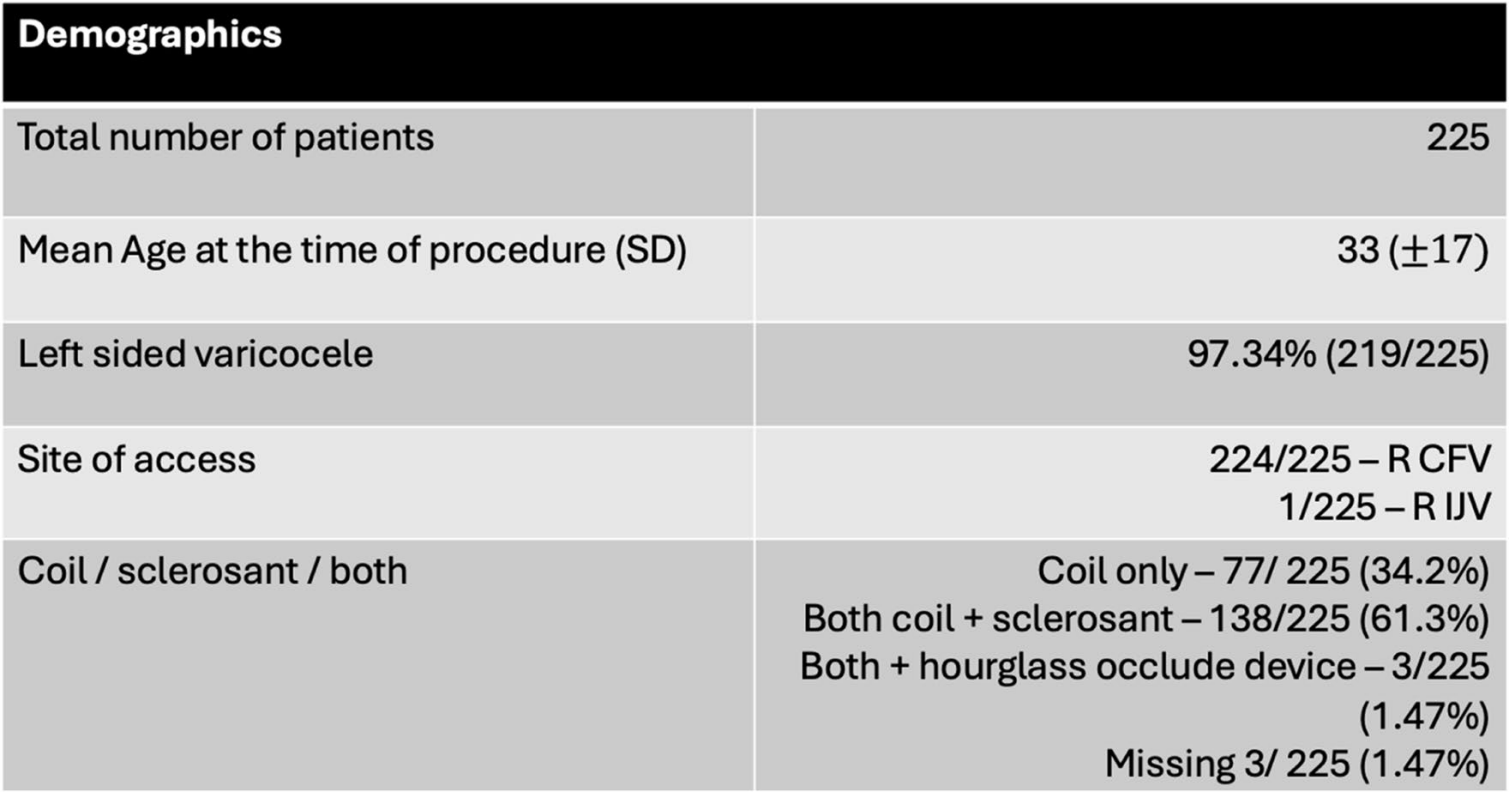
Telephone consultation questionnaire

Patients were treated with either coil embolization alone or coil embolization and sclerosant (Sodium Tetra Decryl 1% diluted 3/1 with air). In limited cases, both coil embolization and an Hourglass™ occlusion device were utilized. Inguinal ring compression was not applied.

Our primary endpoints were the technical and clinical success rates of percutaneous varicocele embolization, as defined above. Our secondary endpoints included recurrence rates measured on telephone consultations and repeat ultrasounds. We also compared technical and clinical success and recurrence rates for the different embolic agents.

Limitations include a lack of semen or hormonal analysis data for analyzing fertility outcomes, the lack of a control group and potential operator bias as a single operator performed all procedures. We have obtained ethical approval for this project from our local ethics committee.

## Results

A cohort of 225 male patients were included in the study. The mean age of patients at the time of the procedure was 33 years $(\pm17)$. Most patients, 97.34% (219 out of 225), had a left-sided varicocele. Vascular access was obtained via the right common femoral vein (RCFV) in almost all cases (224 out of 225 patients). In a single case, access was achieved through the right internal jugular vein (RIJV). Patient demographics are demonstrated in Table 2.

**Table 2:**
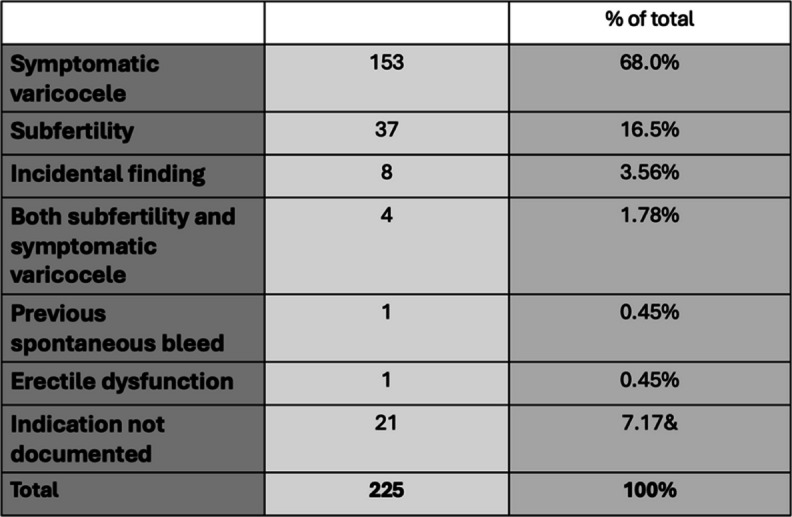
Demographic and procedural characteristics of male patients undergoing varicocele embolization

The indications for embolization are outlined in Table 3. The most common indication for varicocele embolization was the presence of a symptomatic varicocele (scrotal pain or discomfort), accounting for 68% (153/225) of the cases. Subfertility was the second most frequent reason for embolization, representing 16.5% (37/225) of the patients. A smaller percentage (3.56%, 8/225) underwent the procedure following an incidental finding of a varicocele, while 1.78% (4/225) had both subfertility and symptomatic varicocele. Rare indications included a history of previous spontaneous bleeding and erectile dysfunction, each accounting for 0.45% (1/225). In 7.17% (21/225) of cases, the indication for embolization was not documented.

**Table 3:**
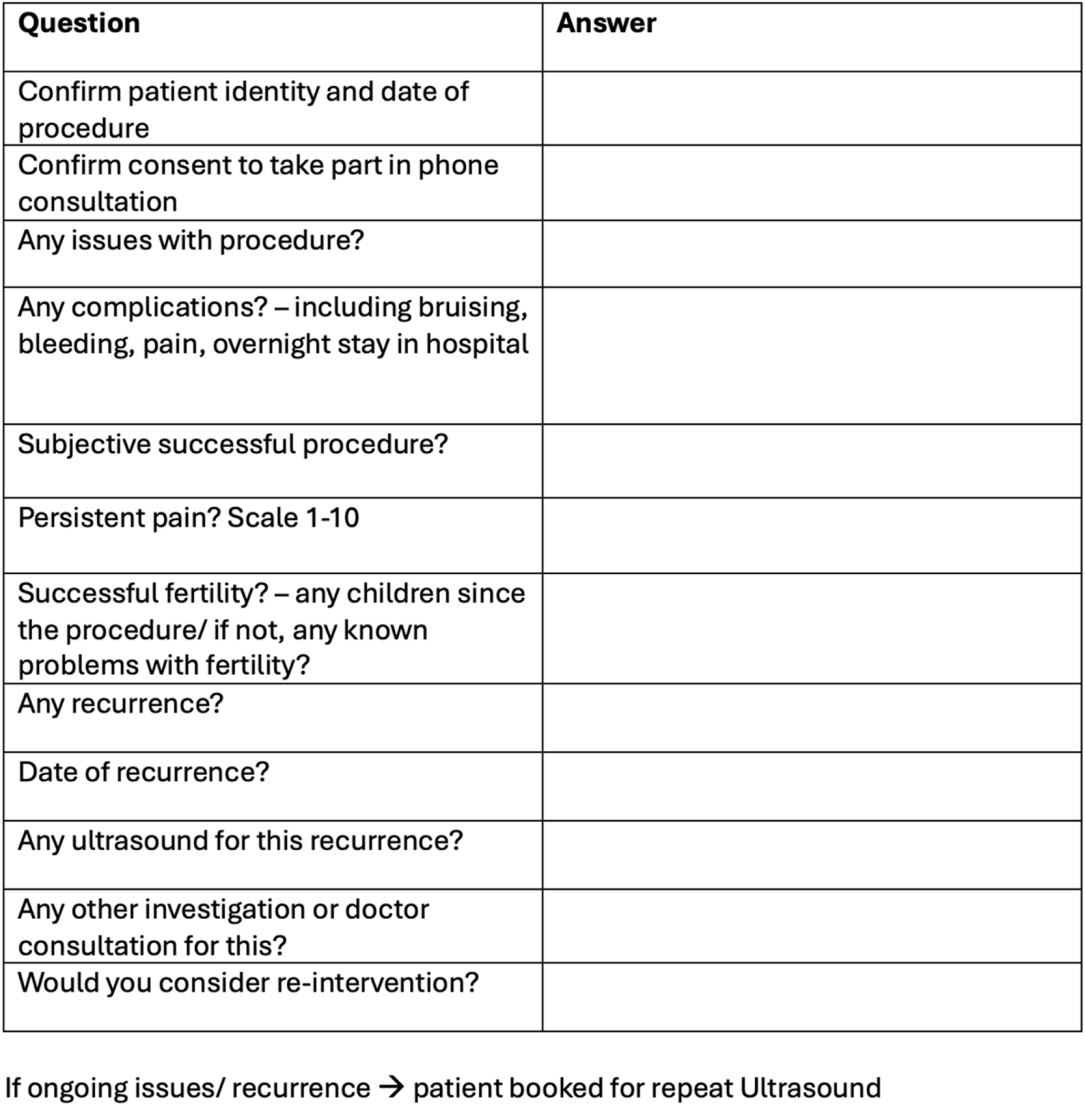
Indications for varicocele embolization in our patient cohort

### Technical success

The technical success rate of embolization procedures in this cohort was 96% (216/225). Of those unsuccessful, 22.2% (2/9) underwent successful repeat procedures, resulting in an overall technical success rate of 96.9%. The remaining seven cases that were not technically successful did not proceed to surgical or further interventional radiology procedures. Of the technically successful cases, 93.75% achieved clinical success on telephone follow-up. There was no association between the side of varicocele or site of access and technical success rate (*p* = 1.0, *p* = 0.0117). A subset of patients (3.12%, 7/225) had previously failed surgical interventions (all left-sided varicoceles), with one patient having undergone two unsuccessful surgeries; all of these patients were successfully treated with interventional radiology (IR), with no recurrences reported. However, 0.89% (2/225) required surgical intervention despite repeated IR attempts (all left-sided varicoceles).

### Complications

The complication rate was 1.78% (4/225). According to the CIRSE classification system for complication reporting, there were two grade 1a complications (oversedation, which required reversal and successful retrieval of coil inserted into left renal vein), one grade 2 complication (groin haematoma which required an overnight stay) and one grade 4 complication (reported mild persistent pain 2/10) [15].

### Type of embolization device used

Of the total, 34.2% (77 patients) underwent coil embolization alone, while 61.3% (138 patients) received a combination of coil embolization and a sclerosing agent. A small subset (1.47%, three patients) were treated with coil embolization alongside an Hourglass™ occlusion device. Treatment modality data were unavailable for 1.47% of patients.

There was no statistically significant association between the type of embolic agent used and either technical or clinical success (*p* = 0.201 and *p* = 0.056, respectively). Similarly, no significant relationship was found between the embolic agent and recurrence rates or the need for re-intervention (*p* = 0.056 and *p* = 1.0, respectively). Although no recurrences were observed in the group treated with the Hourglass™ occlusion device, the sample size was too small to allow for definitive conclusions. Furthermore, there was no significant association between the type of embolic agent used and successful fertility outcomes (*p* = 0.021).

### Telephone follow-up and fertility

This study achieved a telephone consult response rate of 42.7% (96/225) for follow-up of patient-related outcomes. Among the 37 patients for whom fertility was a primary concern, 51.35% (19/37) reported successful fertility following the procedure, while 6.9% (4/37) continued to experience fertility issues. The remainder of the patients denied making further attempts at conception. There was no association between the side of varicocele and successful fertility (*p* = 0.473).

### Recurrence

The recurrence rate reported on telephone follow-up was 25% (24/96). We invited the 24 patients who self-reported recurrence for repeat ultrasonography (US) to ascertain the actual recurrence rate. Twelve patients attended for their repeat ultrasounds, with 50% (6/12) demonstrating true recurrence. Two of these (2/6, 33.33%) were unsuitable for repeat embolization due to their small size. Four were offered repeat embolization procedures, to which they consented. The actual recurrence rate following ultrasound evaluation of patients who self-reported recurrence on telephone follow-up was 6.25% (6/96). The mean follow-up duration of 8.03 ± 3.92 years and a minimum follow-up of 19 months. The mean time to recurrence was 34 ± 17.7 months.

## Discussion

Our technical success rate of 96% is comparable with published meta-analysis data, which have demonstrated 86.1% to 100% success rates [16]. Our study's high clinical success rate of 93.75% on telephone and ultrasound follow up reinforces the efficacy of this intervention, confirming its role as an excellent treatment option for patients presenting with symptomatic varicocele.

Our cohort's initial reported recurrence rate according to telephone follow up was 25%. However, using telephone consultation and repeat ultrasounds, the actual recurrence rate was much lower at 6.25%. This observation is impressive, considering our mean follow-up was 8.03 years $(\pm 3.92)$ with a minimum follow-up of 18 months. Our study's mean time to recurrence was 34 $(\pm17.7)$ months. A Cochrane review in 2021 reported recurrence rates of 13% [7]. Interestingly, both Persad et al. and Jing et al. reported no difference in recurrence between those treated surgically and those treated with embolization [7, 17].

The complication rate in this cohort was lower than similar previous reported studies (1.78%), supporting the established safety profile of embolization compared to surgical alternatives [17]. Halpern et al. reported a 3–3.7% incidence of epididymitis with percutaneous embolization, a 10% risk of haematoma and an up to 12% risk of post-embolic hydrocele [18–21]. In our study, none of the patients experienced severe complications such as testicular loss, a known risk of surgical intervention. Studies have shown that patients receiving IR treatment have a significantly lower risk of adverse events than those undergoing surgical treatment (RR 0.63) [11]. Our study strengthens the evidence that percutaneous embolization offers a safer alternative to varicocelectomy.

Economic studies have demonstrated that surgical embolectomy and percutaneous embolization costs are comparable [22, 23]. However, the potential advantage for those undergoing percutaneous embolization compared to surgical treatment lies in the shorter recovery time to full activity [24]. Patients treated with percutaneous embolization often return to work after 48 h, whereas those treated surgically are advised to avoid strenuous activity for at least three weeks [25]. Dewire et al. noted that 24% of surgical patients required an overnight hospital stay, while all patients treated percutaneously were discharged on the same day [22].

In cases of varicocele embolization failure, several underlying mechanisms may be implicated, including incomplete venous occlusion, collateral vessel recruitment, or missed alternative venous drainage pathways. A repeat embolization procedure should be considered in technically feasible cases, particularly where persistent reflux is demonstrated. CT venography can aid in identifying residual or alternative venous channels not visualized during the initial intervention [25]. Technical challenges typically arise in cases of varicoceles with venous collaterals that bypass competent valves in the cranial segment of the internal spermatic vein, or in the presence of right-sided varicoceles [25, 26]. Late failure is thought to be due to the opening of persistent small venous collaterals into the internal spermatic vein [27]. A structured approach incorporating imaging and tailored re-intervention may enhance long-term clinical outcomes in these cases.

Our findings demonstrate no significant association between the type of embolic agent used and technical success, clinical success, recurrence rates, need for re-intervention, or fertility outcomes. These results suggest that adding a sclerosing agent to coil embolization does not confer a measurable clinical advantage. Although no recurrences were observed in the small group treated with the Hourglass™ occlusion device, the limited sample size precludes definitive conclusions. These observations support using coil embolization alone as a sufficient and effective treatment modality. This is interesting economically as the addition of sclerosant was not of benefit. Replicate findings were previously reported in a systematic review by Makris et al. [14]. Additionally, they concluded that at 1 year, glue was the optimal agent, with coils being the second most effective. Glue had the lowest recurrence rates, and sclerosants alone had the highest recurrence rates (4.2% vs 11.03%) [14].

Our study also highlighted the success of embolization in patients with prior failed surgical interventions. All seven patients with previous unsuccessful surgeries (including one patient who had undergone two attempted surgical repairs) were successfully treated by IR without recurrence. This finding is particularly important as it underscores the value of embolization as a salvage therapy for patients who have failed conventional surgical treatments.

Fertility outcomes are a critical consideration in varicocele treatment, particularly given the association between varicoceles and male infertility. Of the 37 patients in whom fertility was the primary concern, 51.35% achieved successful fertility (live birth) post-procedure. Nabi et al. previously demonstrated successful fertility rates of 40%; however, the follow-up in this study was only 3.6 years [28]. In our study, 6.9% of patients continued to have fertility issues. Improvement in semen parameters following varicocele embolization is well documented [29]. A Cochrane review published in 2004 indicated that the number needed to treat (NNT) for varicocele repair to improve fertility outcomes is 7 [2]. Our study demonstrated higher successful fertility rates than previous studies; however, this may be affected by our longer follow up period.

In conclusion, our data confirms that varicocele embolization is a highly effective and durable treatment option with a low complication rate, even in patients who have undergone prior failed surgical intervention. The high success rate in improving fertility in sub-fertile men also reinforces embolization as a valuable intervention in this patient population. The choice of embolic material did not significantly influence any assessed outcomes, suggesting no added benefit from adding a sclerosing agent to coil embolization.

## Data Availability

The datasets used and/or analysed during the current study are available from the corresponding author on reasonable request.
